# Fabrication and nanostructure control of super-hierarchical carbon materials from heterogeneous bottlebrushes†
†Electronic supplementary information (ESI) available: Experimental details and additional information about material characterization. See DOI: 10.1039/c6sc03961h



**DOI:** 10.1039/c6sc03961h

**Published:** 2016-11-24

**Authors:** Yeru Liang, Luyi Chen, Dongyang Zhuang, Hao Liu, Ruowen Fu, Mingqiu Zhang, Dingcai Wu, Krzysztof Matyjaszewski

**Affiliations:** a Materials Science Institute , PCFM Lab and GDHPPC Lab , School of Chemistry and Chemical Engineering , Sun Yat-sen University , Guangzhou 510275 , P. R. China . Email: wudc@mail.sysu.edu.cn; b Department of Chemistry , Carnegie Mellon University , 4400 Fifth Avenue , Pittsburgh , PA 15213 , USA; c College of Materials and Energy , South China Agricultural University , Guangzhou 510642 , P. R. China

## Abstract

Advances in the performances of many modern materials fundamentally depend upon the exploitation of new micro/nanostructures. Therefore, ingenious design of hierarchical structures through the mimicking of natural systems is of increasing importance. Currently, there is an urgent need for creation of multidimensional carbonaceous structures by integrating a customized hierarchical pore architecture and hybrid carbon framework. Here we report the pioneering fabrication of novel super-hierarchical carbons with a unique carbonaceous hybrid nanotube-interconnected porous network structure by utilizing well-defined carbon nanotube@polystyrene bottlebrushes as building blocks. Hypercrosslinking of such heterogeneous core–shell structured building blocks not only allows for constructing amorphous microporous carbon shells on the surface of graphitic carbon nanotube cores, but also leads to formation of covalently interconnected nanoscale networks. Benefiting from such a well-orchestrated structure, these super-hierarchical carbons exhibit good electrochemical performances. Our findings may open up a new avenue for fabrication of unique and unusual functional carbon materials which possess well-orchestrated structural hierarchy and thus generate valuable breakthroughs in many applications including energy, adsorption, separation, catalysis and medicine.

## Abstract 2

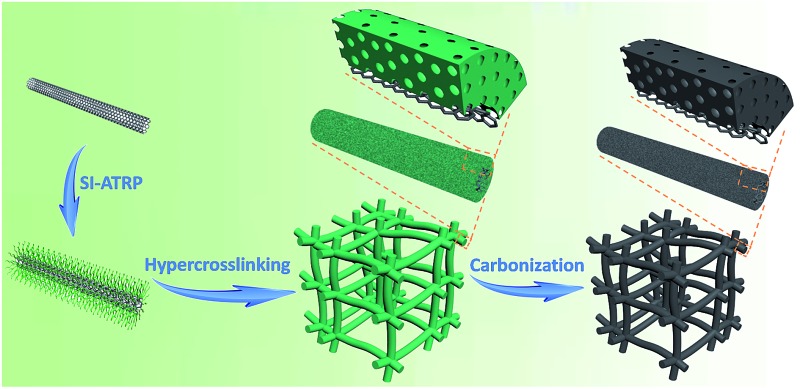
Super-hierarchical carbons with a unique carbonaceous hybrid nanotube-interconnected porous network were fabricated by utilizing well-defined carbon nanotube@polystyrene bottlebrushes as building blocks.

## Introduction

Nature has created numerous examples of intriguing materials with different functionalities, which is an important source of inspiration for materials design and other associated disciplines.^1–6^ The structural hierarchy in these materials plays a vital role in contributions to their exceptional performances. Compared to conventional materials exhibiting a unitary (non-hierarchical) structure, new materials with multiple levels of structural hierarchy can provide positive synergies between each organization, thus leading to multiple benefits including valuable improvements in mechanical, transport, responsive and other properties.^7–10^ As one of these new materials, novel carbon materials with hierarchical porosity or frameworks are particularly appealing. This is motivated by their exceptional electrical conductivity, high surface area, and excellent physicochemical stability. Potential applications range from energy storage, catalysis, gas adsorption to molecular separation.

With the recent development of nanotechnology, significant advances have been attained in construction of hierarchical pore structures for hierarchical carbons.^11–21^ However, these current hierarchical carbons with various hierarchical pore structures generally possess a purely amorphous framework. In addition, there are rare examples of hybrid carbons with an amorphous/graphitic framework, but these carbon materials lack a hierarchical micro–meso–macroporous structure.^22–30^ Moreover, explicit control over the nanoscale and mesoscale architectures of hierarchical carbons remains challenging. Therefore, well-controlled integration of well-defined hierarchical pore architecture and hybrid carbon framework into hierarchical carbons remains a very challenging but desirable nanostructure design.

**Fig. 1:**
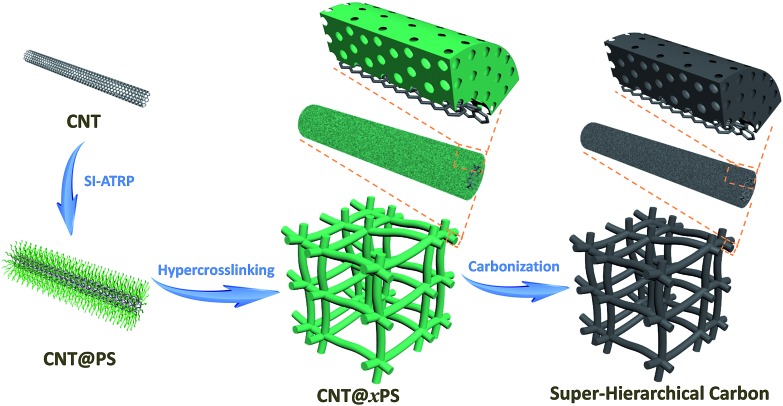
Schematic illustration of the preparation of super-hierarchical carbons from well-defined heterogeneous core–shell CNT@PS bottlebrushes.

Herein we present an unprecedented and controllable protocol for a pioneering construction of a class of novel super-hierarchical carbons (SHCs) with a unique carbonaceous hybrid nanotube-interconnected porous network structure. The key to this advanced protocol is a new heterogeneous core–shell structured building block. The building block selected to demonstrate this procedure is a class of well-defined carbon nanotube@polystyrene (CNT@PS) bottlebrushes. As illustrated in Fig. 1, CNT@PS bottlebrushes are initially prepared by grafting PS chains from the surface of CNT *via* surface-initiated atom transfer radical polymerization (SI-ATRP). Then CNT@PS bottlebrushes are treated through a facile hypercrosslinking reaction to provide the PS shell with well-developed microporosity, thus forming porous organic/inorganic hybrid nanonetworks CNT@crosslinked PS (CNT@*x*PS). SHCs are obtained after a direct carbonization treatment of CNT@*x*PS. The as-constructed SHC materials possess unusual super-hierarchies by delicately integrating a hybrid carbon framework with a hierarchical porous nanonetwork structure. Given the benefits of controllable adjustment of PS chains at the molecular level *via* SI-ATRP, the nanostructures of SHCs can be well tuned. The full use of the excellent electrical conductivity of the CNT cores, the well-developed microporosity within the carbon shells as well as the fast mass transport pathway of the interconnected meso-/macroporous nanonetwork structure led to much superior material properties, as exemplified by their greatly improved energy storage performances.

## Results and discussion

### Preparation of super-hierarchical carbons

**Fig. 2:**
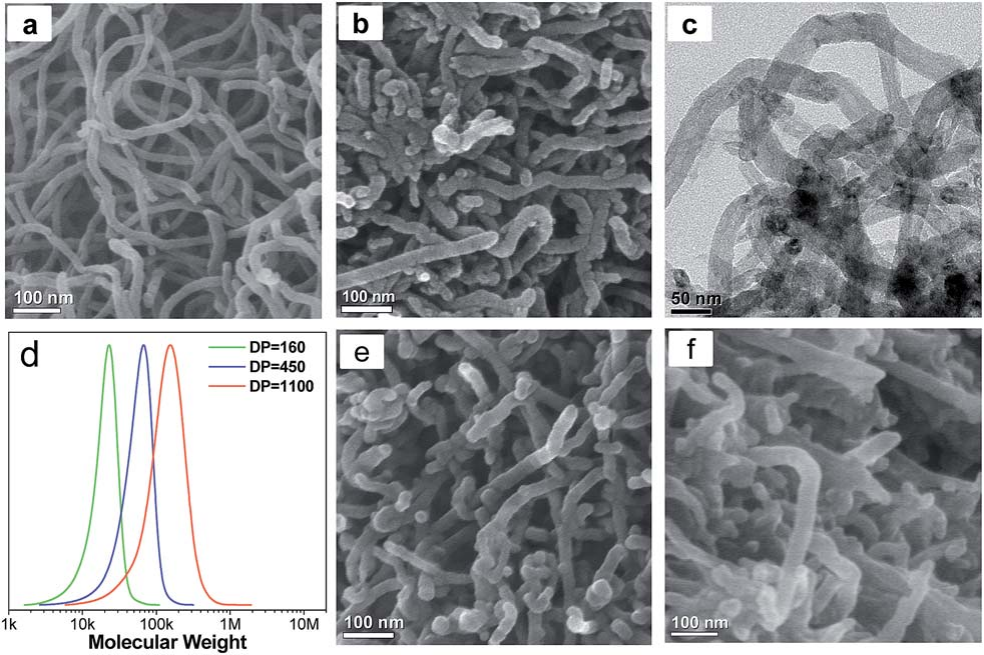
(a) SEM image of CNT-1. (b) SEM and (c) TEM images of CNT@PS_450_ bottlebrushes. (d) GPC trace of the free PS chains, *i.e.*, PS_160_ (green), PS_450_ (blue) and PS_1100_ (red). The *M*_w_/*M*_n_ of PS_160_, PS_450_ and PS_1100_ are 1.24, 1.26 and 1.38, respectively. SEM images of (e) CNT@PS_160_ and (f) CNT@PS_1100_ bottlebrushes.

The heterogeneous core–shell CNT@PS bottlebrushes were prepared by grafting well-defined PS chains from the surface of CNTs *via* the SI-ATRP strategy (Fig. 2a). First of all, the Br-containing surface ATRP initiation sites are introduced onto the surface of CNTs by reacting 2-bromo-2-methylpropionyl bromide with hydroxyl groups functionalized CNTs (Fig. S1†).^31^ According to the results of the thermal gravimetric analysis (TGA), the density of Br atoms on the surfaces of the resulting CNT-1–Br is measured to be 0.186 mmol g^–1^ (Fig. S2†). Subsequently, the SI-ATRP grafting of PS from CNT-1–Br is carried out. As shown in the SEM image of Fig. 2b, the obtained CNT@PS bottlebrushs (*i.e.*, CNT@PS_450_) presented an average diameter of 31 nm. Taking into account the diameter of the pristine CNTs (*i.e.*, 21 nm), the thickness of the grafted PS shell was calculated to be about 5 nm (Fig. 2c and S3†). As measured by TGA, the PS content of CNT@PS_450_ was 36 wt% (Fig. S4†). To estimate the molecular weight of grafted PS chains, a free initiator was added to the reaction system to produce free PS. As shown in the gel permeation chromatography (GPC) trace in Fig. 2d, the obtained free PS_450_ chains have a unimodal peak with molecular weight *M*_n_ = 47 000 and *M*_w_/*M*_n_ = 1.26. It should be noted that due to the physical agglomeration, the CNT@PS bottlebrushes entangle with each other to form a 3D nanonetwork, which facilitates the subsequent hypercrosslinking reaction of the PS chains (Fig. 2b).

**Fig. 3:**
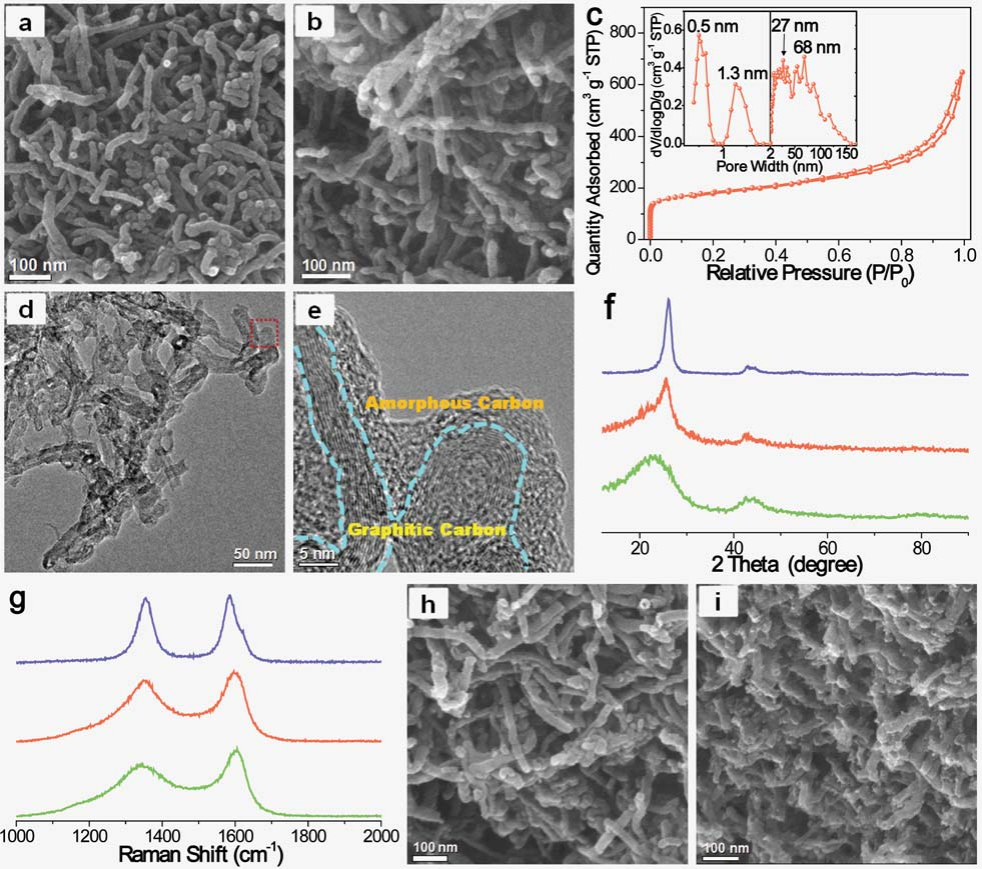
SEM images of (a) CNT@*x*PS_450_ and (b) SHC-450. (c) N_2_ adsorption–desorption isotherm of SHC-450. The inset shows its pore size distribution curve determined by density functional theory (DFT). (d) TEM image and (e) high-resolution TEM image of SHC-450; (e) corresponds to the area indicated by a red rectangle in (d). (f) XRD patterns of CNT-1 (blue), SHC-450 (red) and HPC (green). (g) Raman spectra of CNT-1 (blue), SHC-450 (red) and HPC (green). SEM images of (h) SHC-160 and (i) SHC-1100.

The hypercrosslinking of CNT@PS bottlebrushes was carried out after adding the mixture of anhydrous aluminum chloride and carbon tetrachloride (CCl_4_) under stirring (Fig. 1). The PS chains in the shell of CNT@PS_450_ were swollen in CCl_4_ and then underwent both intra- and interbrush hypercrosslinking by the formation of CCl_2_ crosslinking bridges between the phenyl rings. Subsequently, the CCl_2_ crosslinking bridges were converted into –CO– groups by hydrolysis. During this process, the intrabrush hypercrosslinking subdivided the original solid PS shell into numerous micropores.^32^ The presence of the micropores was confirmed by an adsorption uptake at the low relative pressure (*P*/*P*_0_) in the N_2_ adsorption–desorption isotherm of the resulting CNT@*x*PS_450_ (Fig. S5†). Meanwhile, the interbrush hypercrosslinking helped the PS chains on the periphery of the shell to interpenetrate and then covalently interconnect with each other, eventually leading to formation of a highly stable 3D nanonetwork structure (Fig. 3a). The diameter of the crosslinked bottlebrush nanonetwork unit for CNT@*x*PS_450_ was about 28 nm (Fig. S3c†). The close and loose hypercrosslinking aggregations of network units led to formation of numerous mesopores and macropores, respectively (Fig. 3a and S5†). The BET surface area and micropore surface area were 423 and 100 m^2^ g^–1^, respectively (Table S1†).

The targeted SHC was obtained by carbonization of CNT@*x*PS at 900 °C. The SEM image of the as-prepared SHC-450 clearly demonstrates that the 3D nanonetwork morphology was well retained after harsh carbonization (Fig. 3b). It should be noted that unlike the conventional CNT nanonetwork resulting from the physical agglomeration, the carbonized bottlebrush nanonetwork units of the SHC-450 covalently interconnect with each other to form a highly stable 3D nanonetwork structure, due to the interbrush hypercrosslinking characteristic of its precursor CNT@*x*PS_450_. The diameter of the nanonetwork unit was reduced to about 26 nm by analyzing the SEM image (Fig. S3d†), because of mass loss of non-carbon elements and carbon-containing compounds during carbonization. As shown in the N_2_ adsorption–desorption isotherm in Fig. 3c, the adsorption amount increased very sharply at low *P*/*P*_0_, indicating the existence of micropores; and after that, the adsorption amount increased gradually but still did not reach a plateau near 1.0 *P*/*P*_0_, demonstrating the presence of mesopores and macropores. By comparing the pore size distribution (PSD) curves of the samples before and after carbonization treatment, the carbonization led to the formation of numerous micropores of 0.5 nm. Furthermore, the mesopores and macropores throughout the nanonetwork ranging from 2 to 150 nm had maximum PSD peaks at 27 and 68 nm, respectively (the inset in Fig. 3c). The BET surface area was measured to be up to 635 m^2^ g^–1^, and the micropore surface area and meso-/macropore surface area were 287 and 348 m^2^ g^–1^, respectively (Table S2†). The total pore volume was as high as 1.00 cm^3^ g^–1^.

As illustrated in the TEM image of Fig. 3d, the nanonetwork unit of SHC-450 was mainly composed of microporous carbon and CNTs, and the microporous carbon shell was uniformly coated onto the surface of the sidewall of the CNTs. The high-resolution TEM image (Fig. 3e) clearly depicted the amorphous carbon shell with abundant micropores tightly connected with the distinct graphitic layer of CNT core. In contrast, no obvious micropores could be found in the CNTs (Fig. S6†), and no distinct graphitic structures were observed in the hierarchical porous carbon (HPC) control sample from PS_450_ instead of CNT@PS_450_ (Fig. S4 and S7†). X-ray photoelectron spectroscopy (XPS) analysis indicates that SHC-450 contains a proper amount of oxygen (6.2 at%), which is incorporated in its amorphous carbon shell framework (Fig. S8†). The hierarchical characteristic of the carbon framework was further determined by X-ray diffraction (XRD) pattern and Raman spectrum. The wide-angle XRD pattern of SHC-450 exhibits one sharp and one weak diffraction peaks, corresponding to the (002) and (101) diffractions of the graphitic structure, respectively (Fig. 3f); and its decreased (002) diffraction intensity relative to the pristine CNTs was caused by the shield effect of the microporous carbon shells to X-rays. In the Raman spectrum in Fig. 3g, all samples showed two characteristic peaks around 1350 (D-band) and 1600 cm^–1^ (G-band). Comparatively, the calculated *I*_D_/*I*_G_ ratio for SHC-450 is 1.9, a value that is in between those for CNT (1.0) and HPC (2.4). Based on these measurements, their carbon framework structure parameters could be obtained, as shown in Tables S3 and S4.† A comparison of the interlayer spacing (*d*_002_), stack height (*L*_c_) and stack width (*L*_a_) intuitively highlights that SHC-450 indeed possessed a hybrid carbon framework combined by the graphitic structure of CNT and the amorphous structure of microporous carbon.

### Nanostructure control of super-hierarchical carbons

Our preparation method developed here allows for control over the nanostructures of SHCs at the molecular level.^33,34^ For example, SHCs could be well tailored by variation of the molecular structure of CNT@PS bottlebrushes. Thus, an increase in the degree of polymerization (DP) of the PS side chains of CNT@PS bottlebrushes from 160 to 1100 (Fig. 2d–f and S9†) resulted in a growth of the diameter of nanonetwork units from ∼24 nm for SHC-160 to ∼31 nm for SHC-1100 (Fig. S10†). Thus the thicknesses of microporous carbon shell increased from ∼1.5 nm for SHC-160 through ∼2.5 nm for SHC-450 to ∼5 nm for SHC-1100. Moreover, the aggregation of nanonetwork units of SHCs became closer and closer with increasing the DP of PS side chains, because the long PS side chains facilitated the interbrush entangling and then the interbrush hypercrosslinking. As a result, the maximum of PSD for meso-/macropores among the nanonetwork of SHCs decreased (Fig. S11†). Meanwhile, with increasing the DP of the PS side chains from 160 to 1100, the PS contents of CNT@PS bottlebrushes increased from 32 wt% to 45 wt% (Fig. S12†); therefore, the BET surface areas of the SHCs rapidly increased from 223 m^2^ g^–1^ for SHC-160 to 810 m^2^ g^–1^ for SHC-1100 (Table S2†), and the values of (*L*_c_) and (*L*_a_) of SHCs gradually decreased (Fig. S13 and S14, Tables S3 and S4†).

The nanostructures of SHCs can also be readily adjusted by altering the carbonization conditions, including carbonization time and heating rate. The carbonization time was found to play an important role in tuning the BET surface areas of SHCs (Fig. S15–S18†). For example, the BET surface areas increased drastically from 364 to 1122 m^2^ g^–1^ with extending the carbonization time from 1 to 20 h (Fig. S18†), while further increasing the carbonization time to 25 h decreased the BET surface areas to 700 m^2^ g^–1^. In addition, increasing the heating rate from 2 to 10 °C min^–1^ led to a decline in the BET surface areas from 614 to 530 m^2^ g^–1^ (Fig. S19†). The 3D nanonetwork morphology was well retained in various SHCs in spite of their distinct porous structures (Fig. S20 and S21†), indicating the good stability of the nanomorphology during various carbonization treatments.

### Electrochemical supercapacitance performance

**Fig. 4:**
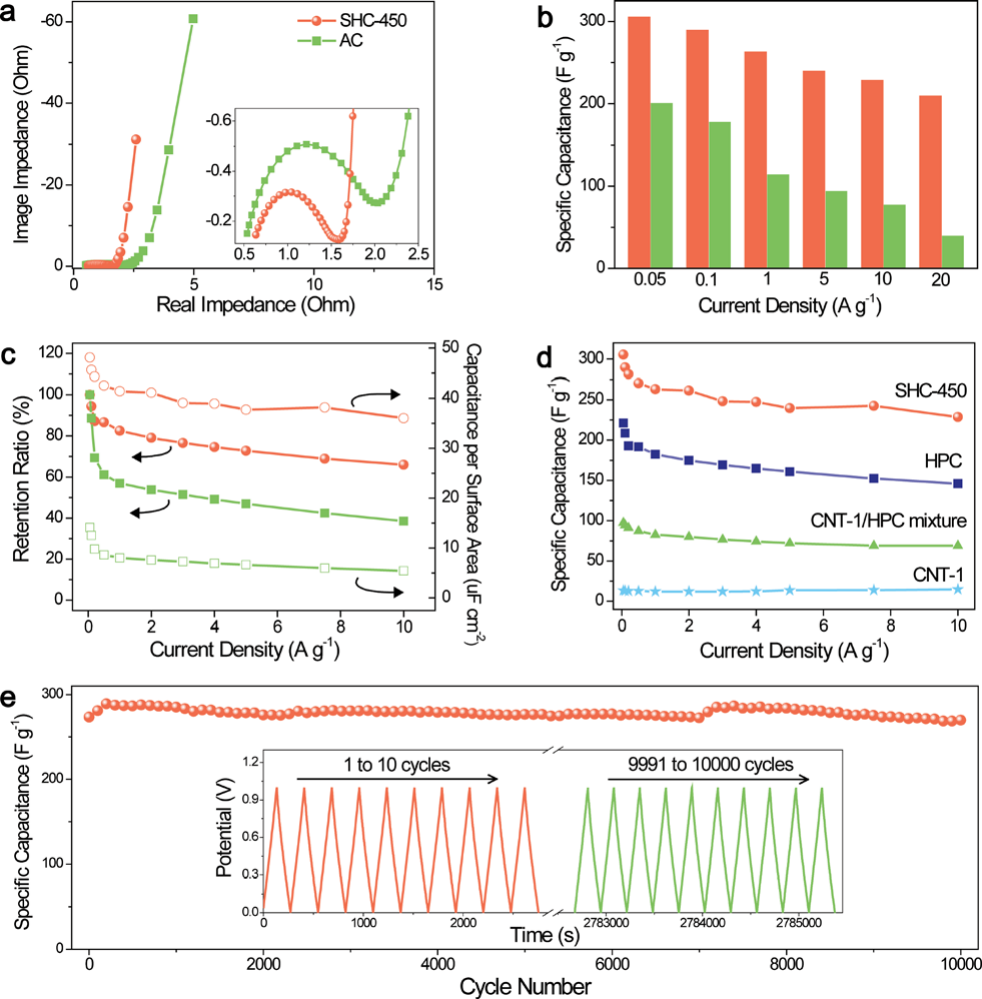
(a) Electrochemical impedance spectra, (b) specific capacitances, (c) capacitance retention ratios and specific capacitances per surface area for SHC-450 (red) and AC (green). (d) Comparison of specific capacitances for SHC-450, HPC, CNT-1/HPC mixture and CNT-1. (e) Long-term cycling stability over 10 000 cycles for SHC-450 at a current density of 1 A g^–1^; the inset shows the curves for the first and last ten cycles.

Benefiting from the well-orchestrated hybrid carbon framework and the adjustable hierarchical nanonetwork structure, the as-prepared SHCs could hold promise for their application in energy storage. Here, we focus on the electrochemical properties of SHC-450 as the electrodes of supercapacitors. For comparison, a high-surface-area activated carbon (AC) is also studied. The Nyquist plots in Fig. 4a show that the diameter of the semicircle for SHC-450 (0.9 ohm) is smaller than that for the AC (1.5 ohm), suggesting that SHC-450 has lower impedance at the electrode/electrolyte interface and faster movement of electrolyte ions inside the nanopores. The resistance for ion diffusion critically influences the capacitive performance, especially for the rate capability of the electrode. For example, the specific capacitance of SHC-450 is as high as 306 F g^–1^ at 0.05 A g^–1^ and retains 240 F g^–1^ even with 100 times increasing of the current density from galvanostatic charge–discharge tests (Fig. 4b). Even increasing the current density to a very high value of 20 A g^–1^, a high capacitance of 210 F g^–1^ still can be obtained. Their capacitances and the corresponding calculated capacitance retention ratios significantly exceed those of AC (Fig. 4c), and many other reported porous carbon electrodes (Table S5†). Furthermore, the specific capacitances per surface area for SHC-450 (36–48 μF cm^–2^) are obviously much higher than that of AC, no matter whether the current density is high or low (Fig. 4c). These values are also clearly higher than those of most porous carbons, indicative of excellent rate capability and extraordinarily efficient electrochemically active surface area (Table S6†). Moreover, after 10 000 cycles, SHC-450 still remains about 100% of the initial capacitance at a high current density of 1 A g^–1^, exhibiting an excellent cycling stability (Fig. 4e).

To gain insight into the important roles of the super-hierarchical structure on the enhanced ion diffusion rate, energy storage capacity and electrochemically active surface, the electrochemical properties of control samples, *i.e.*, CNT-1 and HPC, were investigated. It can be found that CNT-1 has excellent capacitance retention ratios but low specific capacitances, mainly due to its graphitic structure with high conductivity and low specific surface area (Fig. 4d and S22†). Meanwhile, HPC shows high specific capacitances but moderate capacitance retention ratios, resulting from its robust microporous structure but amorphous carbon framework with a relatively low conductivity. In sharp contrast, SHC-450 delivers higher specific capacitances and specific capacitances per surface area than CNT-1 and HPC at various current densities, which obviously indicates that there exists a high synergistic effect between CNT-1 and HPC during the electrochemical charge–discharge procedure (Fig. 4d and S23–S25†). All values of the synergistic effect coefficient (SEC) at different current densities are more than 4.0 for SHC-450, and the maximum SEC is as high as 5.0 when operated at 7.5 A g^–1^ (Fig. S26†). Moreover, simply adding together the capacity contribution of each constituent component (CNT-1 and HPC) would give significantly lower capacitances than the measured capacitances for SHC-450 (Fig. 4d). The values of SEC for SHC-450 under all current densities are higher than those for CNT-1/HPC mixture (Fig. S26†), further highlighting the advantage of the covalent hybrid of the electroactive microporous carbon shell and highly conductive CNT core.

These results demonstrated that the well-defined SHCs can take advantage of each of the hierarchical structure features with a synergistic effect during the electrochemical charging–discharging process. First of all, SHCs possessed a large amount of interconnected micropores, providing abundant active surface areas for ion storage.^35^ More importantly, these active micropores located in the shell of the carbon framework were also well interconnected with mesopores and macropores among the 3D nanonetwork in various directions, which significantly enhanced pore accessibility. In addition, the well-developed meso-/macropores among the nanonetwork provided fast pathways for ion transportation, thus having a low resistance for ion diffusion from the solution to the inner micropores. Moreover, the oxygen-containing surface functional groups in the amorphous carbon shell could not only improve the wettability of the material in the electrolyte (Fig. S8†), but also induce beneficial pseudocapacitance effects through fast faradaic redox reactions, enhancing the capacitance per surface area.^36^ Last but not the least, the graphitic CNTs as the backbone of the nanonetwork provided highly conductive pathways for electrons, favoring the high rate performance.

## Conclusions

In summary, we have demonstrated a successful development of a novel class of SHCs with a heterogeneous carbonaceous nanotube-interconnected network structure based on the utilization of core–shell structured CNT@PS bottlebrushes as building blocks. Cylindrical CNT@PS bottlebrushes were synthesized by SI-ATRP and then crosslinked to interconnect with each other through covalent bonding, followed by carbonization. Tuning of nanostructures of SHCs could be accomplished by variation of the DP of PS side chain or altering the carbonization conditions. The unusual combination of the well-orchestrated hybrid carbon framework and the adjustable hierarchical nanonetwork structure led to much superior material properties, as exemplified by their greatly enhanced supercapacitance performances. Our finding could open the door for molecular level constructing and tailoring of a novel super-hierarchical carbonaceous structure based upon a new application for heterogeneous bottlebrush macromolecules in nanotechnology, and provide a great opportunity to boost the performances of carbon materials to a new stage in a wide variety of fields including energy, adsorption, separation and catalysis.
