# Supplementary material for: Fabrication and nanostructure control of super-hierarchical carbon materials from heterogeneous bottlebrushes
Source: Chem Sci. 2016 Nov 24;8(3):2101–6. doi: 10.1039/c6sc03961h (PMC5407264; doi:10.1039/c6sc03961h)
Supplement: Supplementary file 1 [file SC-008-C6SC03961H-s001.pdf]

Electronic Supplementary Material (ESI) for Chemical Science.
This journal is © The Royal Society of Chemistry 2016

Supporting Information

Fabrication and Nanostructure Control of Super-Hierarchical Carbon Materials from Heterogeneous Bottlebrushes

Yeru Liang,‡[a, c] Luyi Chen,‡[a] Dongyang Zhuang,[a] Hao Liu,[a] Ruowen Fu,[a] Mingqiu Zhang,[a] Dingcai Wu*,[a] and Krzysztof Matyjaszewski[b]

[a] Materials Science Institute, PCFM Lab and GDHPPC Lab, School of Chemistry and Chemical Engineering, Sun Yat-sen University, Guangzhou 510275, P. R. China

[b] Department of Chemistry, Carnegie Mellon University, 4400 Fifth Avenue, Pittsburgh, PA 15213, USA

[c] College of Materials and Energy, South China Agricultural University, Guangzhou 510642, P. R. China

Experimental

1. Sample preparation

Materials. Styrene (St) was purified by passing through a basic alumina column before polymerization. Copper(I) bromide (CuBr) was purified by washing sequentially with acetic acid and ethanol, filtration and drying, and was stored under nitrogen before use. CNT-1 (Chengdu Organic Chemicals Co. Ltd.), CNT-2 (Shenzhen Nanometer Gang Co., Ltd.), Copper(II) bromide ($CuBr_2$; Shanghai Xinbao, AR), N,N,N′,N”,N”-pentamethyldiethylenetriamine (PMDETA; TCI, 98+%), anhydrous aluminum chloride ($AlCl_3$; Aladdin, AR), ethyl 2-bromoisobutyrate (EBiB; Aladdin, 98%) and other chemicals were used as received.

Synthesis of CNT-1-Br. In a typical synthesis, pristine CNT-1 (1.9 g) was added to a mixture of 65% HNO_3 (58 ml) and H_2O (7 ml). After ultrasonication for 30 min, the mixture was

stirred for 27 h at 120 °C under reflux. The product was collected by filtrating, washed with water for several times and dried under vacuum 90 °C overnight, leading to formation of the carboxyl groups functionalized CNT-1 (CNT-1-COOH). Subsequently, CNT-1-COOH was suspended in 30 ml of $SOCl_2$ and stirred at 70 °C for 24 h, to give the carbonyl chloride groups functionalized CNT-1 (CNT-1-COCl). After removing the excess $SOCl_2$ by vacuum, 60 mL of anhydrous glycol was added to CNT-1-COCl and stirred at 120 °C for 48 h. The solid was filtered off and washed efficiently with anhydrous tetrahydrofuran (THF). After drying under vacuum overnight, the hydroxyl groups functionalized CNT-1 (CNT-1-OH) was obtained. Then, CNT-1-OH (1.4 g), $CHCl_3$ (35 mL), 4-dimethylaminopyridine (0.1 g) and triethylamine (1.5 mL) were placed in a flask immersed in an ice/water bath. The flask was sealed and flushed with N_2. A solution of 2-bromo-2-methylpropionyl bromide (0.72 mL) dissolved in anhydrous $CHCl_3$ (15 mL) was added dropwise and the flask was maintained at 0 °C for 3 h and then at room temperature for 48 h. The product was filtered off under vacuum, thoroughly washed with $CHCl_3$ and dried in a vacuum oven overnight, leading to formation of the Br-modified CNT-1 (CNT-1-Br).

Synthesis of CNT-2-Br. In a typical synthesis, pristine CNT-2 (5.7 g) was added to a mixture of 65% HNO_3 (174 ml) and H_2O (21 ml). After ultrasonication for 30 min, the mixture was stirred for 24 h at 120 °C under reflux. The product was collected by filtrating, washed with water for several times and dried under vacuum 90 °C overnight, leading to formation of the carboxyl groups functionalized CNT-2 (CNT-2-COOH). Subsequently, CNT-2-COOH was suspended in 60 ml of $SOCl_2$ and stirred at 70 °C for 24 h, to give the carbonyl chloride groups functionalized CNT-2 (CNT-2-COCl). After removing the excess $SOCl_2$ by vacuum, 120 mL of anhydrous glycol was added to CNT-2-COCl and stirred at 120 °C for 48 h. The solid was filtered off and washed efficiently with anhydrous tetrahydrofuran (THF). After drying under vacuum overnight, the hydroxyl groups functionalized CNT-2 (CNT-2-OH) was obtained. Then, CNT-2-OH (2.8 g), $CHCl_3$ (70 mL), 4-dimethylaminopyridine (0.2 g) and

triethylamine (3.0 mL) were placed in a flask immersed in an ice/water bath. The flask was sealed and flushed with N_2. A solution of 2-bromo-2-methylpropionyl bromide (1.44 mL) dissolved in anhydrous $CHCl_3$ (15 mL) was added dropwise and the flask was maintained at 0 ^oC for 3 h and then at room temperature for 48 h. The product was filtered off under vacuum, thoroughly washed with $CHCl_3$ and dried in a vacuum oven overnight, leading to formation of the Br-modified CNT-2 (CNT-2-Br).

Synthesis of CNT@PS bottlebrushes. CNT@PS bottlebrushes were synthesized according to the following recipe: St/CNT-Br/CuBr/$CuBr_2$/PMDETA/EBiB =2500/1/5/0.4/6/1 (molar ratio). A Schlenk flask was charged with CNT-Br, $CuBr_2$, PMDETA, St and EBiB. After stirring for 0.5 h under gentle N_2 purge, CuBr was added rapidly under N_2 flow. The solution was bubbled with N_2 for another 1 h to remove air completely. After that, the flask was sealed and put into a water bath of 90 °C. The reaction was stopped by opening the flask and exposing the catalyst to air after different times. The mixture was separated by centrifuging. The transparent green solution was passed through a column of neutral alumina and then precipitated into a large excess of methanol, to give the free PS for measuring the molecular weight by GPC. Meanwhile, the black solid was purified by repeated redispersing in THF and centrifuging for several times, until no precipitation could be collected when the liquid was added into an excess of methanol. After drying, CNT@PS bottlebrushes were obtained. The resulting CNT@PS bottlebrushes were denoted as CNT@PS_x, where the x indicates their DP of PS side chains. Among them, CNT@PS_{160}, CNT@PS_{450} and CNT@PS_{1100} were fabraicated from CNT-1-Br, while CNT@PS_{850} was fabraicated from CNT-2-Br.

Synthesis of SHCs. Typically, 5.00 g of anhydrous $AlCl_3$ and 50 mL of CCl_4 were mixed and then heated at 75 °C for 0.5 h with magnetic stirring in a three-neck flask with a condenser. Then, CNT@PS bottlebrushes were well dispersed into 50 mL of CCl_4 and were subsequently transferred to the above mixture, followed by heating at 75 °C for 28 h with magnetic stirring. One hundred milliliters of 1 mol/L HCl was added slowly to the above mixture and then was

heated at 75 °C for 1 h with magnetic stirring. The product was filtered off, washed with acetone, 1 mol/L HCl, and pure water, followed by drying at 80 °C overnight. After that, the resulting CNT@*x*PS was carbonized at 900 °C for 3 h in N_2 flow with a heating rate of 5 °C/min, leading to formation of SHCs. The resulting SHCs were denoted as SHC-*y*, where the *y* indicates the DP of PS side chains of their precusors (*i.e.*, CNT@PS bottlebrushes). For comparison, a carbon control sample, *i.e.*, HPC, was synthesized from PS_{450} instead of CNT@PS_{450}. Its preparation procedure was exactly the same as that of the SHC-450 except that the free PS_{450} was employed as the precursor. Besides, in order to adjust the nanostructures of SHCs, various targeted SHCs were synthesized by carbonizing the CNT@*x*PS_{850} at 900 °C for the desired hours with different heating rates in a furnace under a N_2 flow. The applied carbonization times were varied from 1 to 25 h with heating rate of 2~10 °C/min.

2. Characterization

Stuctual characterization. The microstructure of the samples was investigated by a JSM-6330F scanning electron microscope (SEM) and a FEI Tecnai G2 Spirit transmission electron microscope (TEM). About 100 nanotubes in a SEM image were picked at random, and then a statistical analysis of the diameter distribution was carried out. XRD patterns were recorded on a D-MAX 2200 VPC diffractometer using Cu K radiation (40 kV, 26 mA). Raman measurement was carried out with inVia-Reflex Renishaw Raman system. Macromolecular weight was analyzed with a Waters Breeze gel permeation chromatography (GPC). The thermogravimetric analysis (TGA) was performed under flowing N_2 condition at a heating rate of 20 °C/min. X-ray photoelectron spectroscopy (XPS) measurements were carried out with an ESCALAB250 instrument. N_2 adsorption measurements were carried out using a Micromeritics ASAP 2020 analyzer at 77K. The BET surface area (S_{BET}) was analyzed by Brunauer-Emmett-Teller (BET) theory. The micropore surface area (S_{mic}) was determined by t-plot method, and then the external surface area (S_{ext}) was obtained by subtracting the S_{mic}

from the S_{BET}. The pore size distribution was analyzed by original density functional theory (DFT) combined with non-negative regularization and medium smoothing. The total pore volume (V_t) was calculated according to the amount adsorbed at a relative pressure P/P_0 of about 0.99.

Fabrication and measurements of supercapacitors. The electrodes in the form of round sheet were obtained by pressing a mixture film of 92 wt.% carbon sample and 8 wt.% poly(tetrafluorethylene). Nickel foam and aluminum grid were used as the current collector in the aqueous electrolyte supercapacitor cell and organic electrolyte supercapacitor cell, respectively. 6 mol/L KOH aqueous solution was used as the electrolyte. The electrochemical measurements were characterized with the assembled coin-type supercapacitor. Cyclic voltammetry (CV) was carried out using an IM6ex electrochemical workstation. Galvanostatic charge-discharge behavior was characterized by BT2000 (ARBIN Instruments).

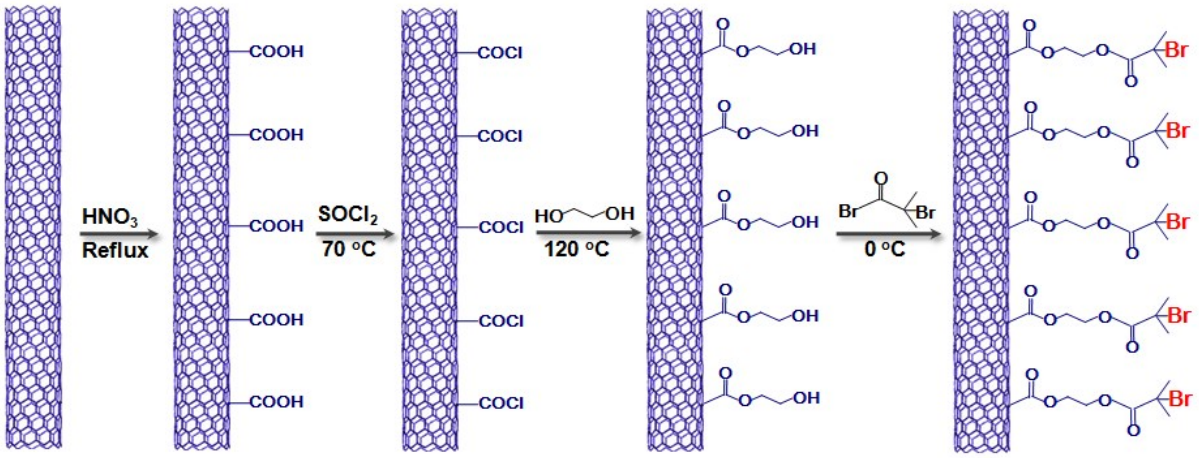

Fig. S1 Schematic representation of the process of introduction of Br-containing surface ATRP initiation sites for CNT. Four steps are included: (1) carboxyl groups were introduced onto the surface of CNT by oxidation of CNT with HNO_3; (2) carbonyl chloride groups functionalized CNT was synthesized *via* reaction of thionyl chloride with carboxyl-contained CNT; (3) hydroxyl groups were introduced onto the surface of CNT by reaction of carbonyl chloride groups functionalized CNT with glycol; (4) Br-modified CNT was obtained by reacting hydroxyl groups functionalized CNT with 2-bromo-2-methylpropionyl bromide.

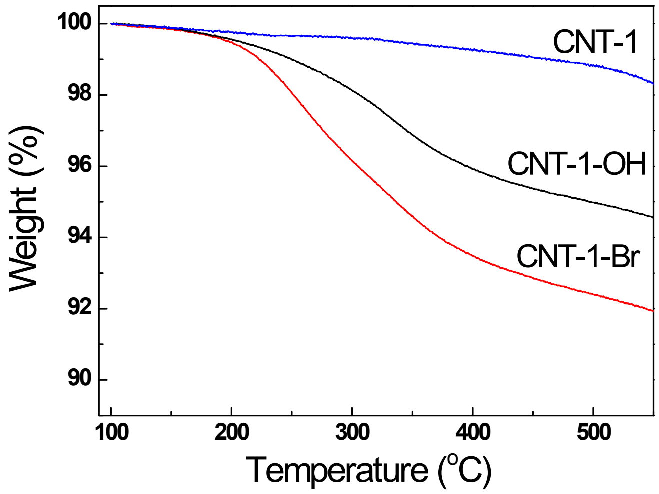

Fig. S2 Thermal gravimetric analysis (TGA) curves of CNT-1, CNT-1-OH and CNT-1-Br. The weight loss of CNT-1, CNT-1-OH and CNT-1-Br below 550 °C is 1.7%, 5.4% and 8.1%, respectively. According to these results, the density of Br atom on the surfaces of the resulting CNT-1-Br is measured to be 0.186 mmol/g.

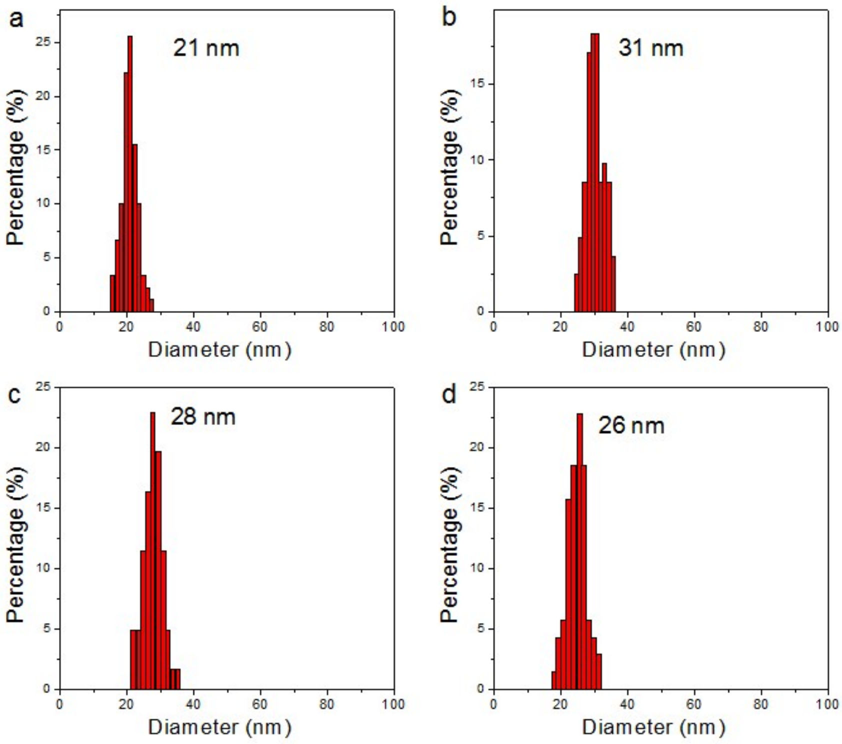

Fig. S3 Diameter distribution histograms from SEM image analysis for (a) CNT-1, (b) CNT@PS_{450}, (c) CNT@$x PS_{450}$ and (d) SHC-450.

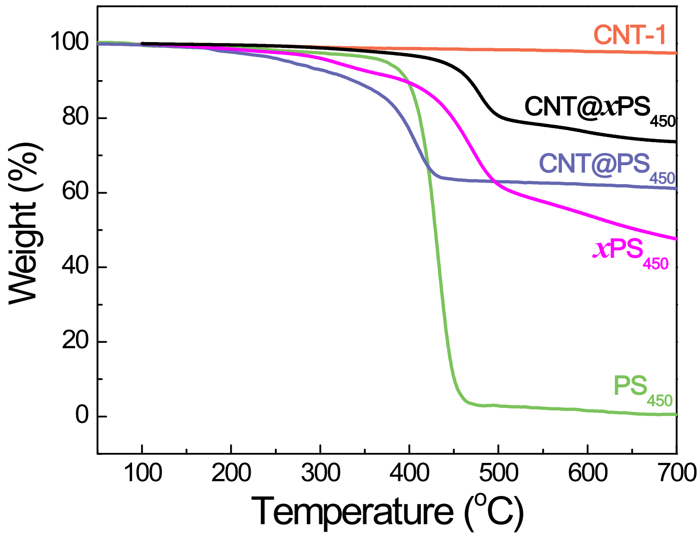

Fig. S4 Comparison of TGA curves of CNT-1, CNT@PS_{450}, CNT@$x PS_{450}$, $x PS_{450}$ and PS_{450} under flowing N_2. The weight loss at 700 °C of CNT-1, CNT@PS_{450}, CNT@$x PS_{450}$, PS_{450} and $x PS_{450}$ are about 3 wt%, 39 wt%, 26 wt%, 100 wt% and 52 wt%, respectively. It can be found that after hypercrosslinking, the carbon yields of *x*PS and CNT@*x*PS are significantly

enhanced, indicating that the hypercrosslinking treatment endows the crosslinking PS framework with good carbonizability.

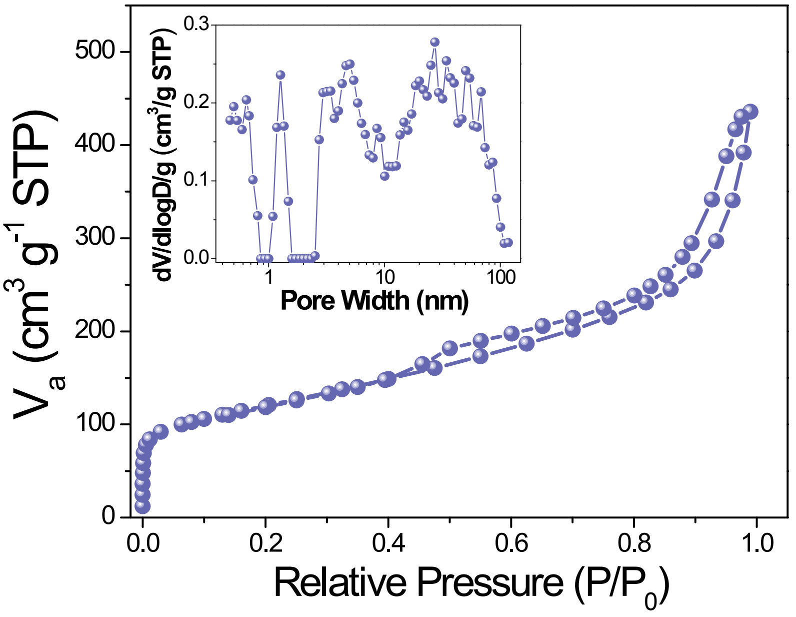

Fig. S5 N_2 adsorption-desorption isotherm of CNT@*x*PS_{450}. The inset shows its pore size distribution.

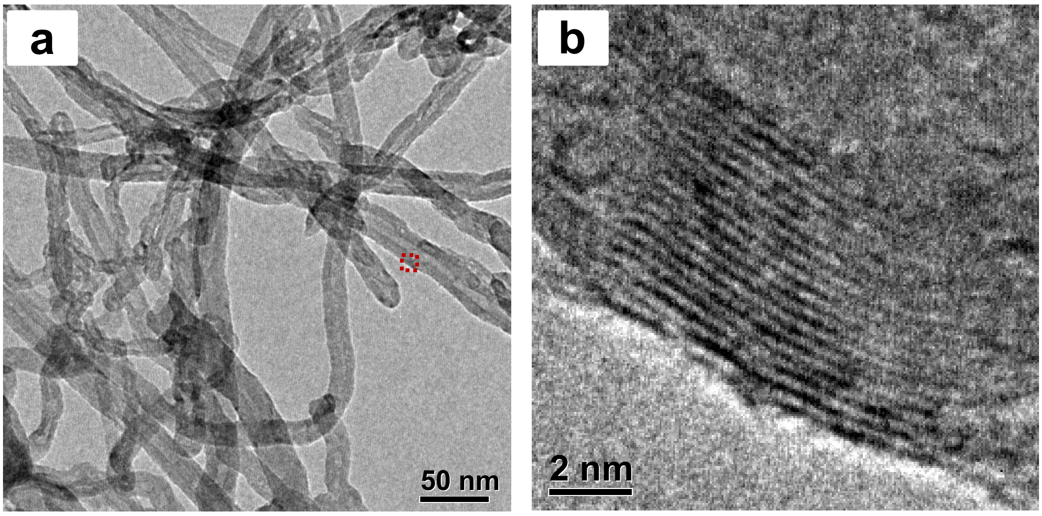

Fig. S6 TEM images of CNT-1. (b) corresponds to the area indicated by a red rectangle in (a).

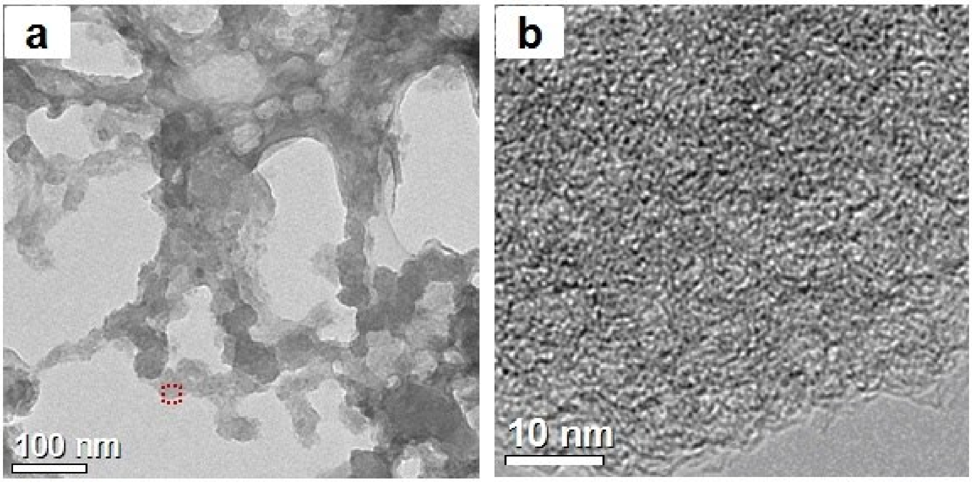

Fig. S7 TEM images of HPC. (b) corresponds to the area indicated by a red rectangle in (a).

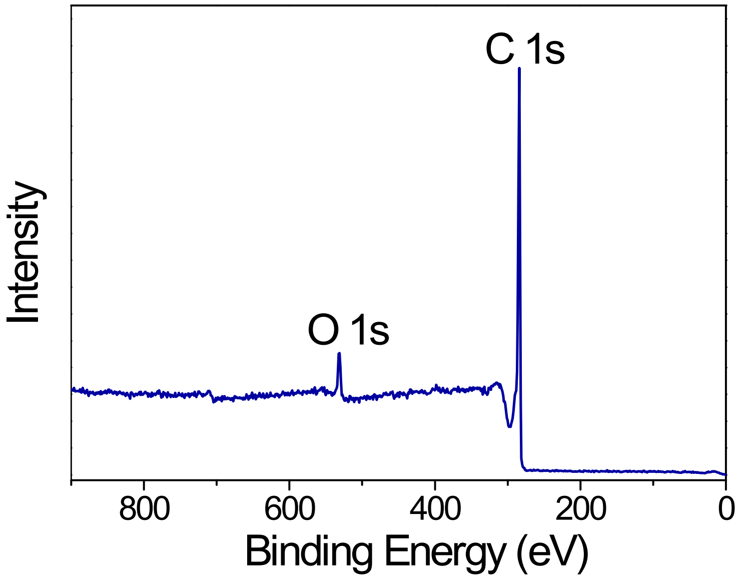

Fig. S8 X-ray photoelectron spectroscopy (XPS) spectrum of SHC-450. XPS analysis detects that only the carbon (93.8 at%) and oxygen (6.2 at%) are present in the carbon framework of SHC-450, without any other impurity. It is known that the hypercrosslinking treatment endows the PS precursor with proper amount of oxygen atom through constructing carbonyl crosslinking bridges between PS chains, which are then transformed into the oxygen-containing surface functional groups after carbonization.

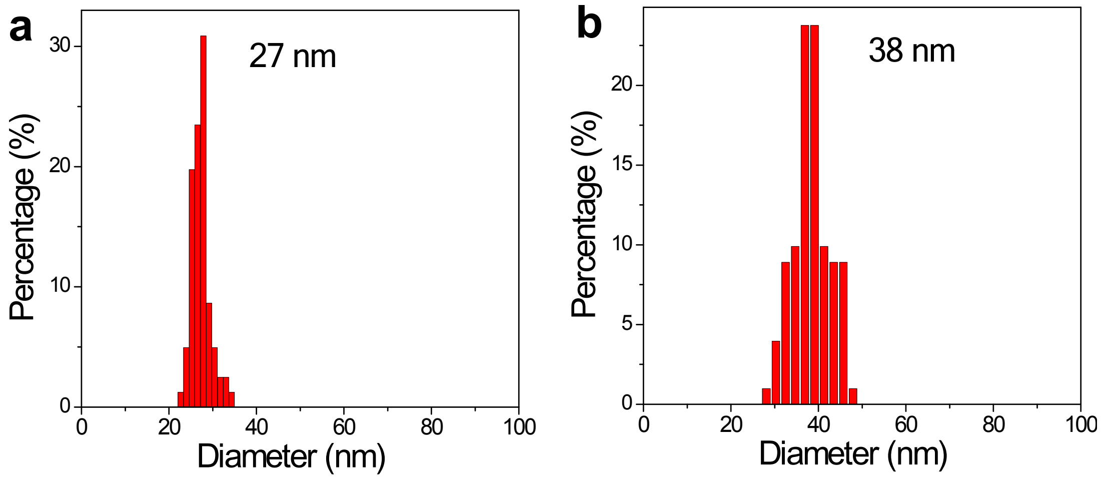

Fig. S9 Diameter distribution histograms from SEM image analysis for (a) CNT@PS_{160} and (b) CNT@PS_{1100}. The diameter of the nanonetwork unit of CNT@PS_{160} and CNT@PS_{1100} are calculated to be ~27 and ~38 nm, respectively. Therefore, an increase in the DP of the PS side chains from 160 to 1100 results in a growth in the thicknesses of PS shell increase from ~3 nm for CNT@PS_{160} and to ~8.5 nm for CNT@PS_{1100}.

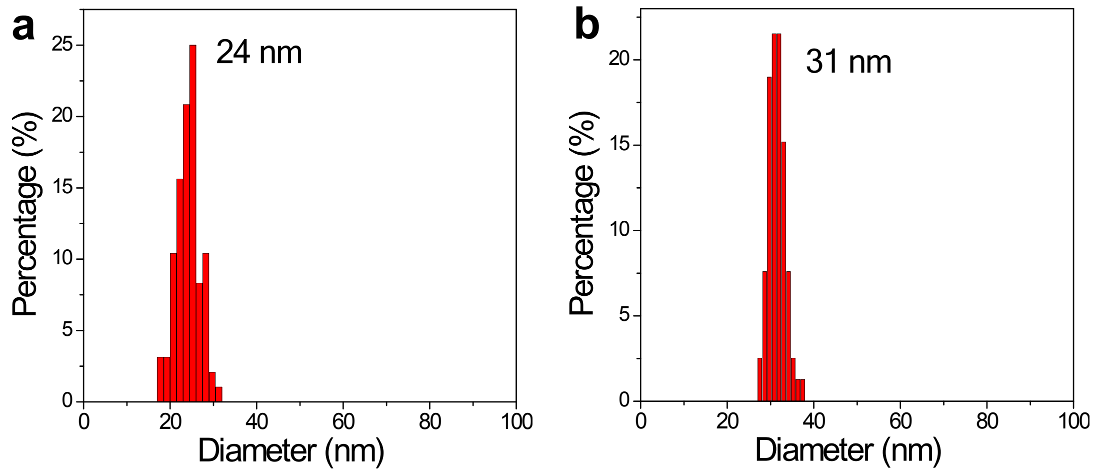

Fig. S10 Diameter distribution histograms from SEM image analysis for the nanonetwork units of (a) SHC-160 and (b) SHC-1100. The diameter of the nanonetwork unit of SHC-160 and SHC-1100 are calculated to be ~24 and ~31 nm, respectively.

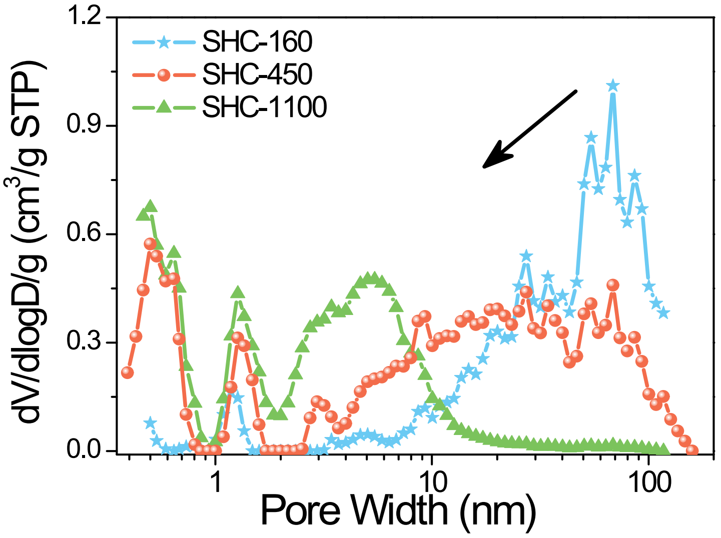

Fig. S11 DFT pore size distribution curves of SHCs. The maximum of PSD decreases from 68 nm for SHC-160 to 27 nm for SHC-450, and to 5 nm for SHC-1100. With increasing the DP of the PS side chains, the maximum of PSD for meso-/macropores among the nanonetwork of SHCs decreases, because of the closer and closer crosslinking aggregation for the network units.

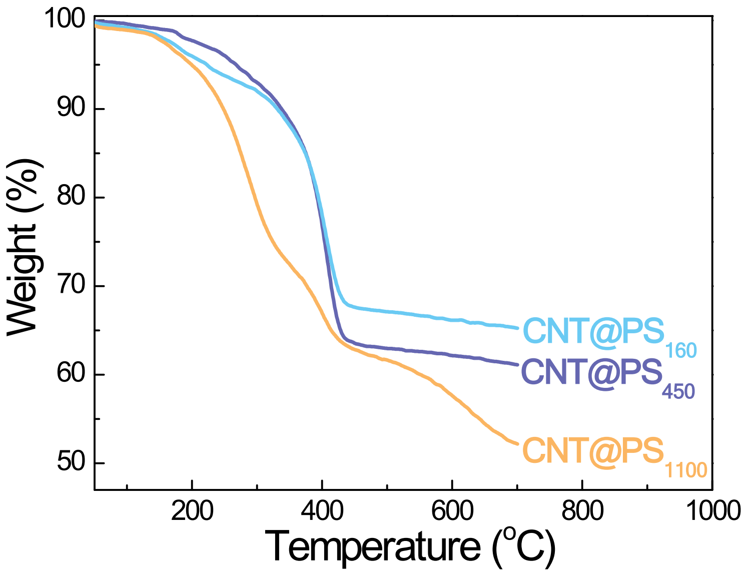

Fig. S12 TGA curves of CNT@PS_{160}, CNT@PS_{450} and CNT@PS_{1100}. The weight loss at 700 °C of CNT@PS_{160}, CNT@PS_{450} and CNT@PS_{1100} are about 35 wt%, 39 wt% and 48 wt%, respectively. Considering the weight loss of CNT at the same condition is about 3 wt% (Fig.

S4), the PS contents of of CNT@PS_{160}, CNT@PS_{450} and CNT@PS_{1100} are 32 wt%, 36 wt% and 45 wt%, respectively.

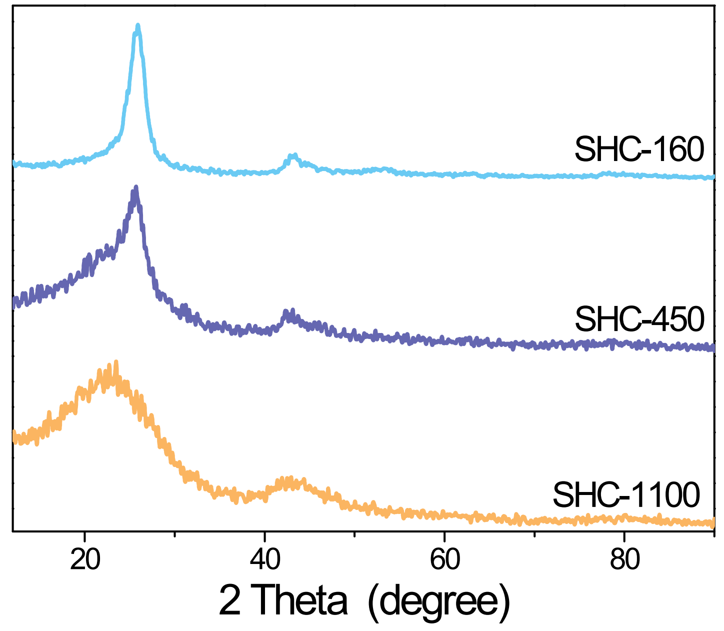

Fig. S13 XRD patterns of SHCs.

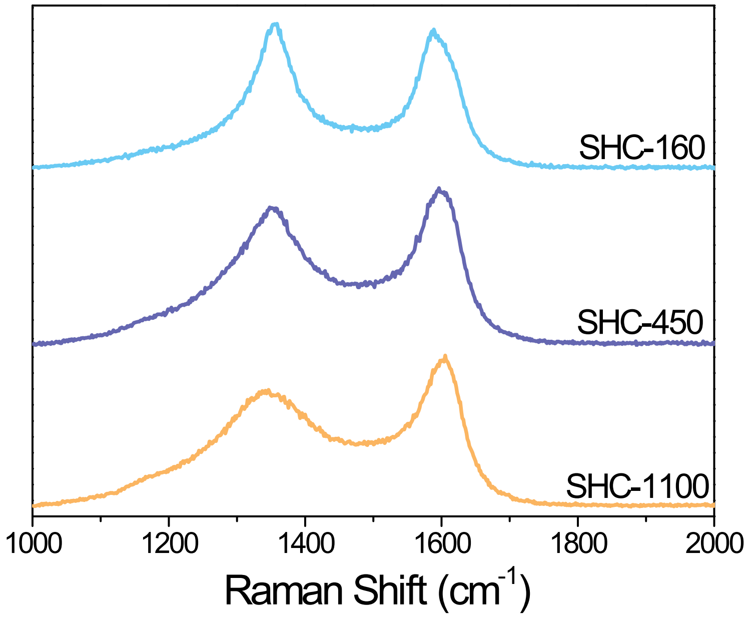

Fig. S14 Raman spectra of SHCs

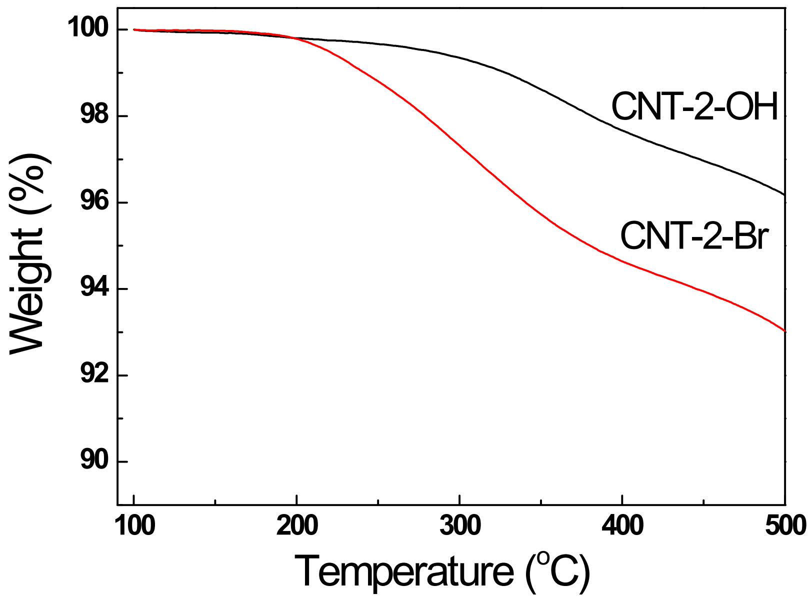

Fig. S15 TGA curves of CNT-2-OH and CNT-2-Br. The weight loss of CNT-2-OH and CNT-2-Br below 500 °C is 3.8% and 7.0%, respectively. According to these results, the density of Br atom on the surfaces of the resulting CNT-2-Br is measured to be 0.215 mmol/g.

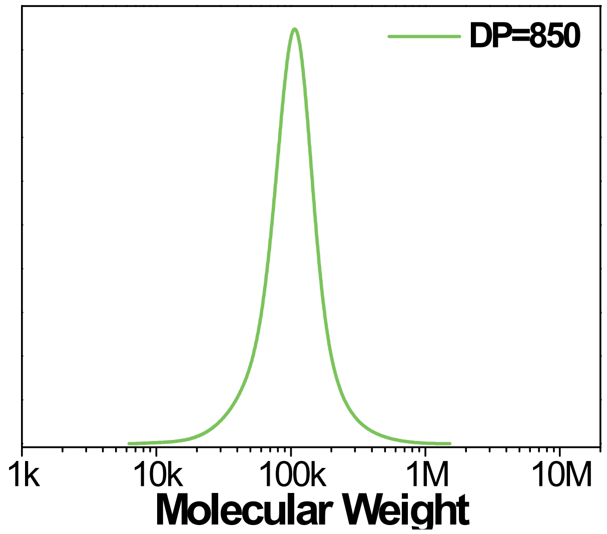

Fig. S16 GPC trace of the free PS_{850} chains. The M_w/M_n of PS_{850} is 1.31.

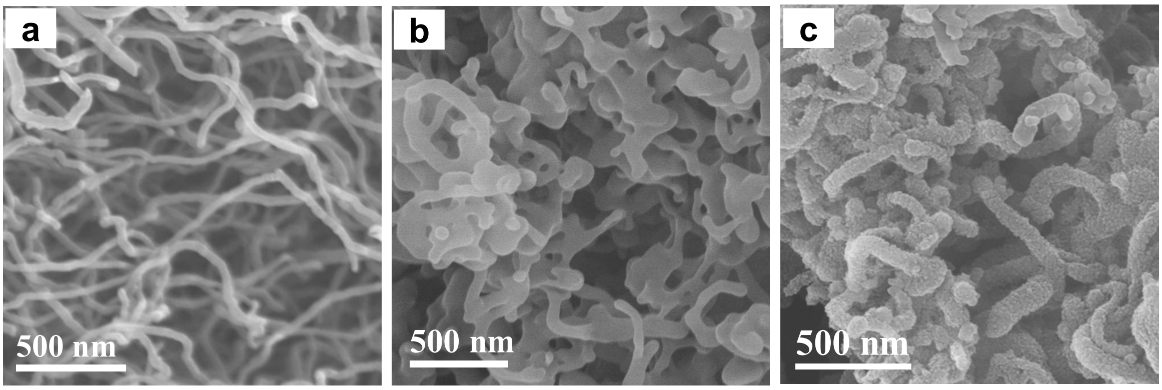

Fig. S17 SEM images of (a) CNT-2, (b) CNT@PS_{850} and (c) CNT@*x*PS_{850}.

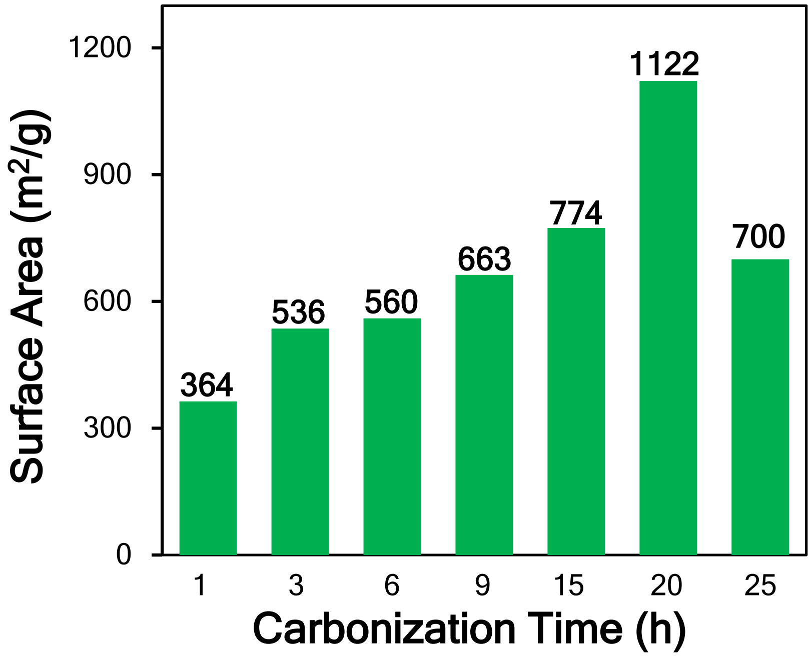

Fig. S18 BET surface areas of SHCs synthesized by carbonizing the CNT@*x*PS_{850} for various carbonization times.

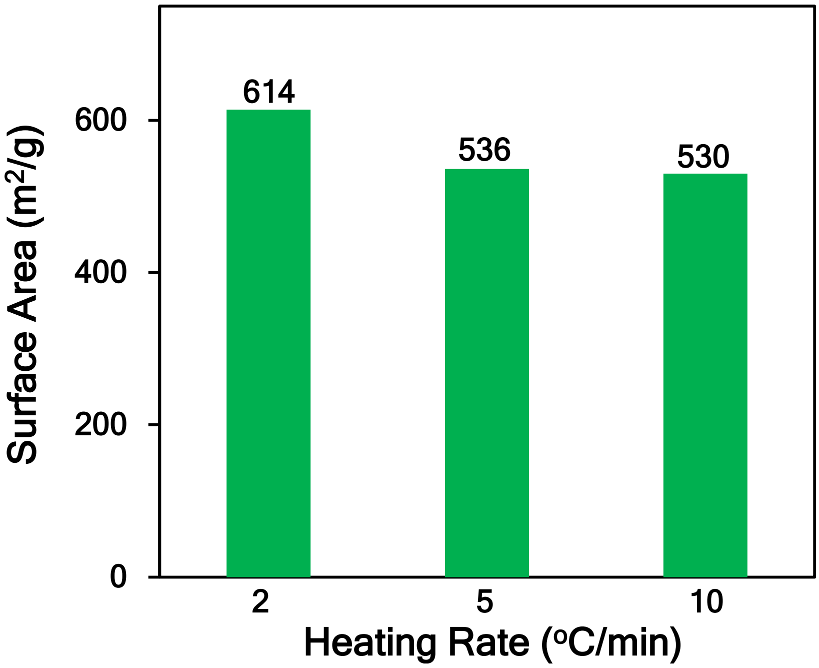

Fig. S19 BET surface areas of SHCs synthesized by carbonizing the CNT@xPS$_{850}$ at various heating rates.

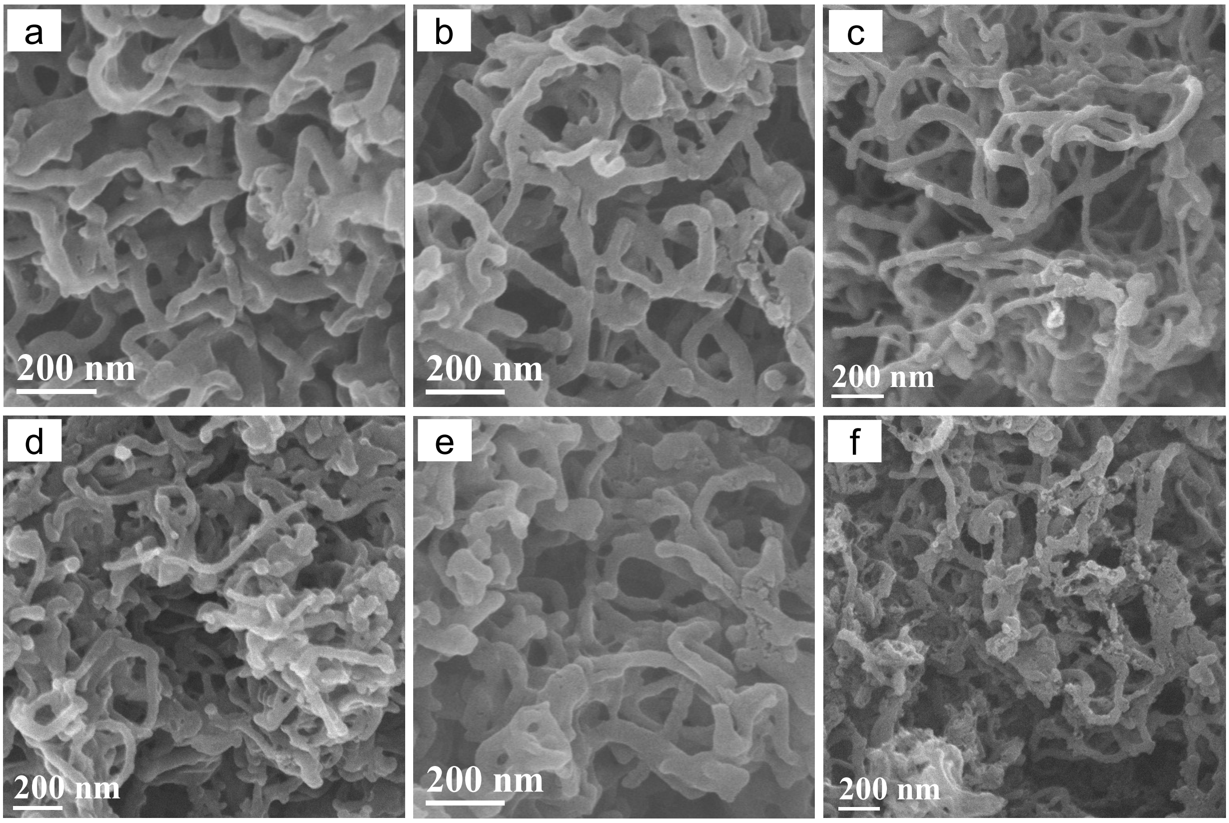

Fig. S20 SEM images of SHCs synthesized by carbonizing the CNT@xPS$_{850}$ for various carbonization times, including (a) 1 h, (b) 3 h, (c) 6 h, (d) 9 h, (e) 12 h and (f) 20 h.

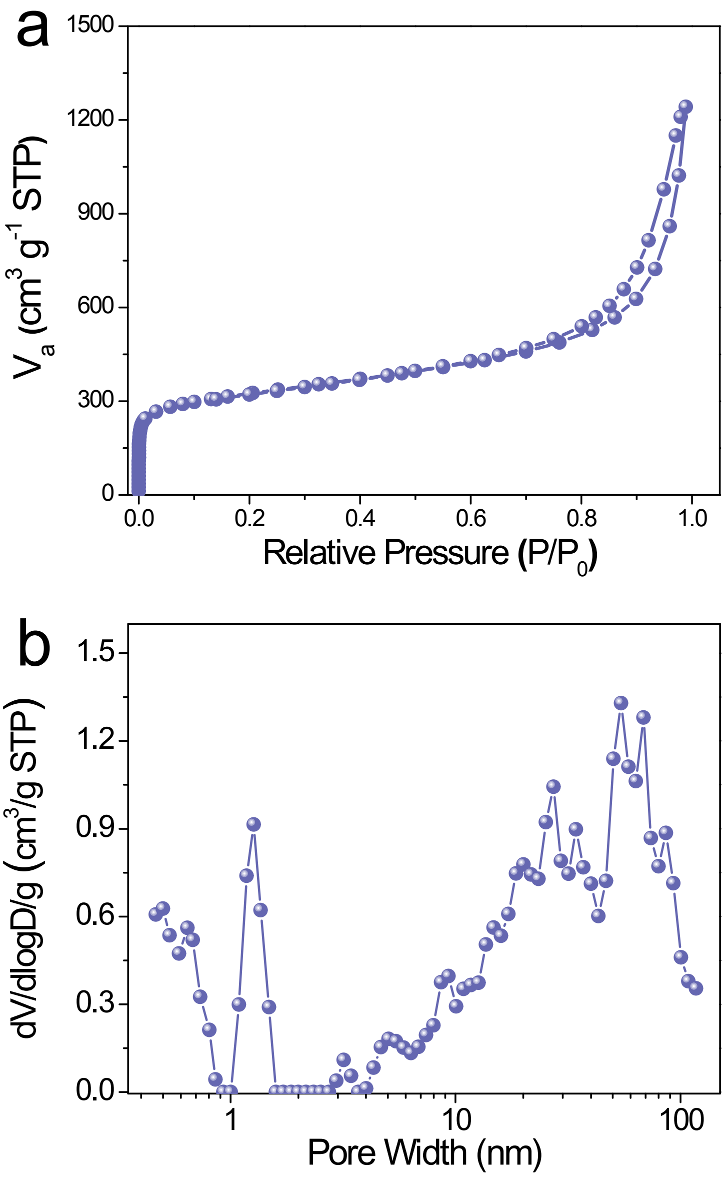

Fig. S21 (a) N_2 adsorption-desorption isotherm and (b) pore size distribution of SHC obtained by carbonizing the CNT@*x*PS_{850} at 900 °C for 20 h with a heating rate of 5 ^{o}C/min.

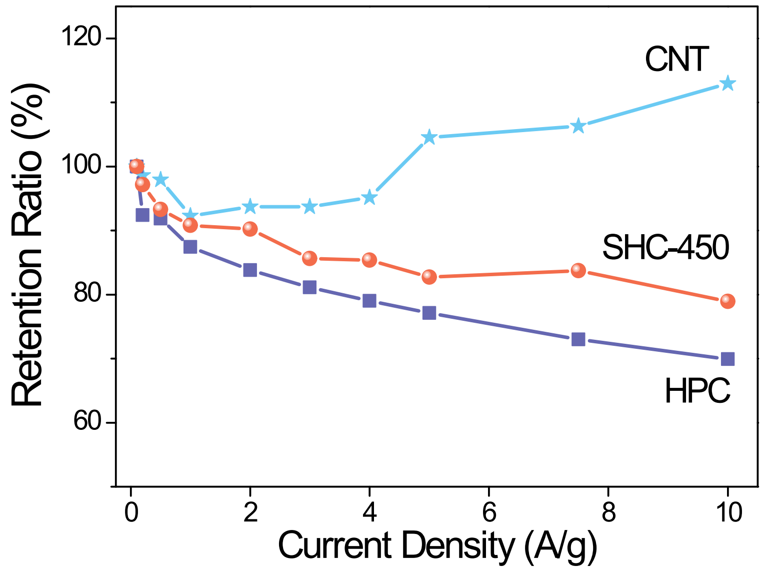

Fig. S22 Capacitance retention ratios of SHC-450, HPC and CNT.

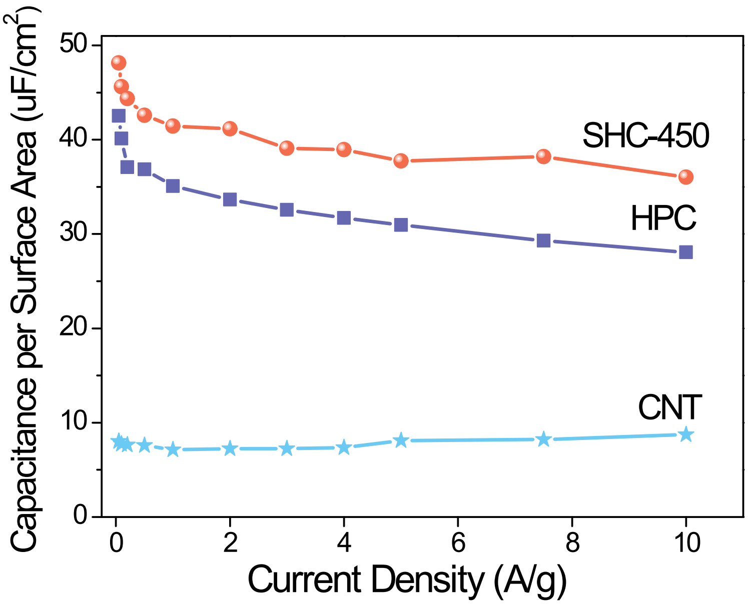

Fig. S23 Specific capacitances per surface area of SHC-450, HPC and CNT at various current densities.

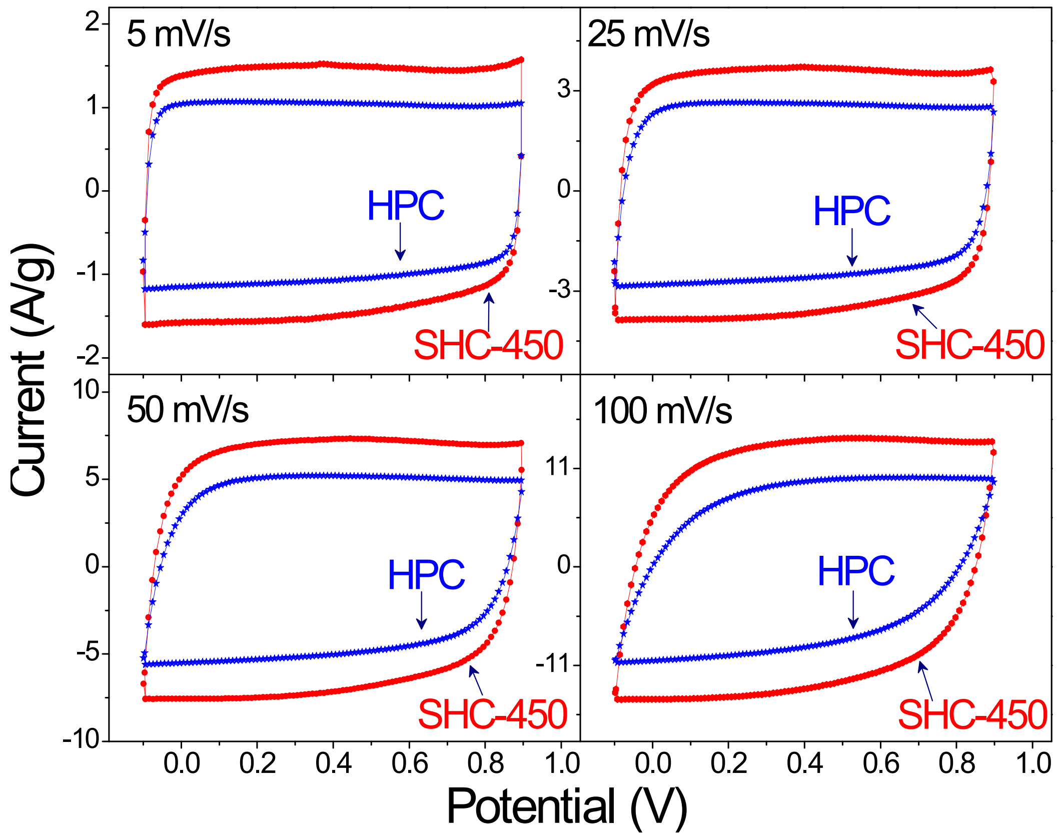

Fig. S24 Cyclic voltammograms (CVs) at various sweep rates for SHC-450 and HPC. Generally, an ideal nanostructure of supercapacitive electrodes can offer fast ion transport pathways and thus the electrical double layer can be re-organized quickly at the switching potentials, exhibiting a rectangle-shaped CV curve.[1] Thus, the ion diffusion behaviors within a nanoporous carbon structure can be estimated by the rectangle degree. The higher the rectangle degree, the better the ion diffusion behavior. At low sweep rates, the electrolyte ions have enough time to move into the nanopores for the EDL formation; therefore, the CV curves present a good rectangular shape for both SHC-450 and HPC at sweep rate of 5 mV/s. But with increasing the sweep rate to 100 mV/s, the rectangle degree of HPC decreases. Comparatively, SHC-450 has much better rectangle-shaped CV curves at high sweep rates such as 100 mV/s as compared to HPC, demonstrating that the ion diffusion rate within the SHC-450 is faster than that within the HPC.

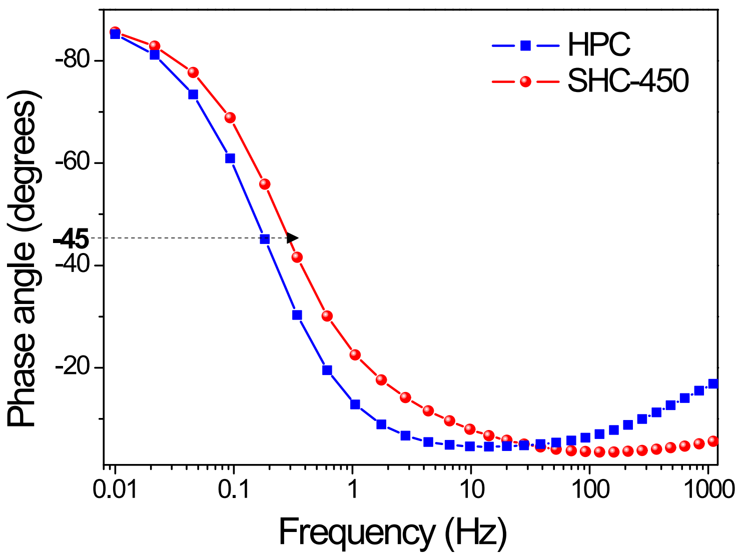

Fig. S25 Impedance phase angle versus frequency for SHC-450 and HPC electrodes. The characteristic frequency f_0 for the –45° phase angle occurs at ~0.3 Hz for the SHC-450 electrode and at ~0.18 Hz for the HPC electrode. This frequency marks the point at which the resistive and capacitive impedances are equal.[2] The corresponding time constant τ_0 (=1/f_0) of SHC-450 equals 3.3 s, which is faster than that of HPC (5.6 s). This rapid frequency response of SHC-450 accounts for its enhanced the ion transport rate in the nanostructure.[3]

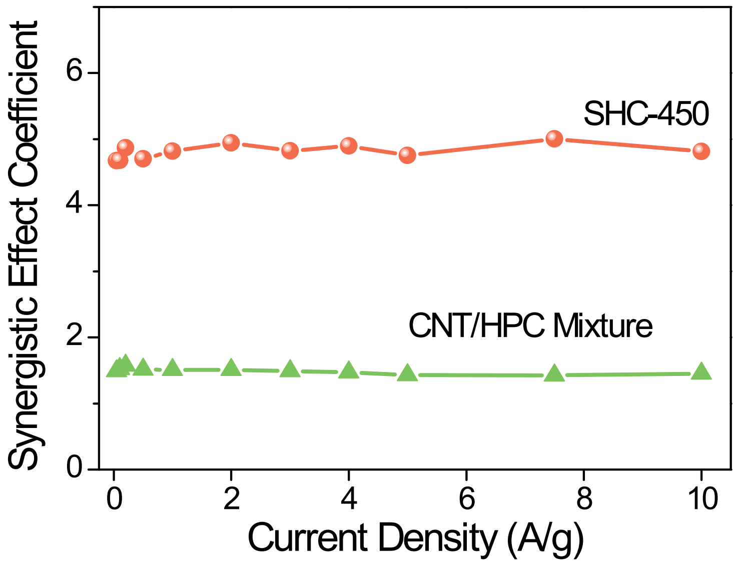

Fig. S26 Synergistic effect coefficients of SHC-450 and CNT/HPC mixture at various current densities.

Table S1 Pore structure parameters of typical samples.

Sample	S_{BET} (m^2/g)	S_{ext} (m^2/g)	S_{mic} (m^2/g)	V_t (cm^3/g)
CNT-1	168	157	11	0.96
CNT@*x*PS_{160}	394	376	18	0.69
CNT@*x*PS_{450}	423	323	100	0.67
CNT@*x*PS_{1100}	479	395	84	0.90

Table S2 Pore structure parameters of typical samples.

Sample	S_{BET} (m^2/g)	S_{ext} (m^2/g)	S_{mic} (m^2/g)	V_t (cm^3/g)
SHC-160	223	153	70	0.74
SHC-450	635	348	287	1.00
SHC-1100	810	430	380	0.59
HPC	520	81	439	0.44

Table S3 Parameters of Raman spectra of typical samples

Sample	Peak area		L_a (nm)	Peak center (cm^{-1})	
	D	G		D	G
CNT-1	114067	114378	4.36	1354	1588
SHC-160	366543	255878	3.04	1356	1590
SHC-450	693847	362260	2.27	1357	1594
SHC-1100	1153450	529040	2.00	1352	1596
HPC	884271	371464	1.83	1360	1598

Table S4 Parameters of XRD paterns of typical samples

Sample	$2\theta_{002}$ (°)	$\cos\theta_{002}$	$\sin\theta_{002}$	β_{002} (°)	d_{002} (nm)	L_c (nm)
CNT-1	26.10	0.974	0.226	1.62	0.341	4.98
SHC-160	25.72	0.975	0.223	2.11	0.346	3.82
SHC-450	25.66	0.975	0.222	3.38	0.347	2.38
SHC-1100	23.80	0.979	0.206	7.7	0.374	1.04
HPC	23.20	0.980	0.201	7.97	0.383	1.01

Table S5 Summary of the capacitances and capactiance retention ratios of representative carbons tested in aqueous electrolyte.

Samples	Refer. Number	Current Density (A/g)	Capacitance (F/g)		Current Density Range (A/g)	Capacitance retention ratio (%)	
			Refer.	This Work		Ref.	This Work
Nitrogen doped ordered mesoporous carbon	4	0.1	219	**290**	0.1~5	49.3	**82.7**
Nitrogen doped ordered nanoporous carbon	5	0.05	292	**306**	0.05~10	45.2	**74.8**
Ordered nanoporous carbon	5	0.05	97	**306**	0.05~10	60.8	**74.8**
Ordered mesoporous carbon (FDU-15)	6	0.5	130	**270**	0.5~20	67.7	**72.4**
Activated FDU-15	6	0.5	199	**270**	0.5~20	62.8	**72.4**
Ordered mesoporous carbon (FDU-16)	7	0.1	34	**290**	0.1~0.5	26.5	**93.1**
Activated FDU-16	7	0.1	260	**290**	0.1~5	73.5	**82.7**
Ordered mesoporous carbon/graphene aerogel	8	0.5	197	**270**	0.5~10	71.6	**84.8**
Graphene	9	0.1	69	**290**	0.1~10	53.6	**79.0**
Nitrogen doped graphene	9	0.1	282	**290**	0.1~10	74.5	**79.0**
Graphene aerogel	8	0.5	130	**270**	0.5~10	65.4	**84.8**
Nitrogen doped graphene-CNT networks	10	0.5	180	**270**	0.5~5	52.8	**88.9**
Microporous carbon nanosphere	11	0.05	176	**306**	0.05~2	62.5	**85.5**
Hollow carbon nanospheres	12	0.1	203	**290**	0.1~10	75.4	**79.0**
Carbon nanocages	13	0.1	260	**290**	0.1~10	68.4	**79.0**
Microporous carbon nanosheets	14	0.5	213	**270**	0.5~10	75.1	**84.8**
Porous carbon nanosheets	15, 16	0.5	241	**270**	0.5~10	53.9	**84.8**
Microporous conducting carbon beehive	17	0.5	254	**270**	0.5~20	64.6	**72.4**
Functional hierarchical porous carbon	18	2	236	**261**	2~10	81.2	**87.7**
Hierarchically ordered porous carbon	19	1	109	**263**	1~10	81.0	**86.9**
Nitrogen doped porous carbon nanofibers	20	0.5	214	**270**	0.5~10	81.8	**84.8**
Nitrogen doped hydrothermal carbons	21	0.1	220	**290**	0.1~4	63.6	**85.2**
Activated carbon (YP-50)	This work	0.05	200	**306**	0.05~20	19.5	**72.4**

Table S6 Summary of the capacitances per surface area of representative carbons tested in aqueous electrolyte.

Samples	Refer. Number	Low urrent density (A/g)	Capacitances per surface area (μF/cm^2)		High current density (A/g)	Capacitances per surface area (μF/cm^2)	
			Refer.	This Work		Ref.	This Work
Ordered mesoporous carbon (FDU-15)	6	0.5	19.7	**42.6**	20	13.3	**33.1**
Activated FDU-15	6	0.5	14.1	**42.6**	20	8.9	**33.1**
Ordered mesoporous carbon (FDU-16)	7	0.1	6.2	**45.6**	0.5	1.6	**42.6**
Activated FDU-16	7	0.1	23.3	**45.6**	5	17.1	**37.7**
Nitrogen doped ordered nanoporous carbon	5	0.05	28.8	**48.1**	10	13.0	**36.0**
Ordered nanoporous carbon	5	0.05	9.4	**48.1**	10	5.7	**36.0**
Mesoporous carbon nanofibers	22	0.5	16.2	**42.6**	5	11.6	**37.7**
Nitrogen doped porous carbon nanofibers	20	0.5	38.1	**42.6**	10	31.1	**36.0**
Carbon nanocages	13	0.1	14.0	**45.6**	10	9.6	**36.0**
Microporous carbon nanosheets	14	0.5	26.9	**42.6**	10	20.2	**36.0**
Porous carbon nanosheets	15, 16	0.5	9.2	**42.6**	10	4.9	**36.0**
Microporous carbon nanosphere	11	0.05	7.8	**48.1**	2	4.9	**41.1**
Ultrahigh-surface-area hollow carbon nanospheres	12	0.1	9.7	**45.6**	10	7.3	**36.0**
Hierarchical lamellar porous carbon	23	0.05	7.4	**48.1**	20	6.0	**33.1**
Hierarchically ordered porous carbon	19	1	20.7	**41.4**	10	16.8	**36.0**
3D micro-porous conducting carbon beehive	17	0.5	19.1	**42.6**	20	12.4	**33.1**
Nitrogen doped hydrothermal carbons	21	0.1	38.5	**45.6**	4	24.5	**39.0**
Activated carbon (YP-50)	This work	0.05	200	**48.1**	10	5.5	**36.0**

References:

1. Liang, Y., Wu, D. & Fu, R. Preparation and Electrochemical Performance of Novel Ordered Mesoporous Carbon with an Interconnected Channel Structure. *Langmuir* 25, 7783-7785 (2009).
2. Taberna, P. L., Simon, P. & Fauvarque J. F. Electrochemical Characteristics and Impedance Spectroscopy Studies of Carbon-Carbon Supercapacitors. *J Electrochem Soc* 150, A292-A300 (2003).
3. El-Kady, M. F., Strong, V., Dubin, S. & Kaner, RB. Laser scribing of high-performance and flexible graphene-based electrochemical capacitors. *Science* 335, 1326-1330 (2012).
4. Cai, T., Zhou, M., Ren, D., Han, G. & Guan, S. Highly ordered mesoporous phenol–formaldehyde carbon as supercapacitor electrode material. *J. Power Sources* 231, 197-202 (2013).
5. Liang, Y., Liu, H., Li, Z., Fu, R. & Wu, D. In situ polydopamine coating-directed synthesis of nitrogen-doped ordered nanoporous carbons with superior performance in supercapacitors. *J. Mater. Chem. A* 1, 15207-15211 (2013).
6. Lv, Y. et al. A comprehensive study on KOH activation of ordered mesoporous carbons and their supercapacitor application. *J. Mater. Chem.* 22, 93-99 (2012).
7. Wang, Z., Zhou, M., Chen, H., Jiang, J. & Guan, S. Hierarchical Activated Mesoporous Phenolic-Resin-Based Carbons for Supercapacitors. *Chemistry – An Asian Journal* 9, 2789-2797 (2014).
8. Liu, R. et al. An Interface-Induced Co-Assembly Approach Towards Ordered Mesoporous Carbon/Graphene Aerogel for High-Performance Supercapacitors. *Adv. Funct. Mater.* 25, 526-533 (2015).
9. Jeong, H. M. et al. Nitrogen-doped graphene for high-performance ultracapacitors and the importance of nitrogen-doped sites at basal planes. *Nano Lett.* 11, 2472-2477 (2011).
10. You, B., Wang, L., Yao, L. & Yang, J. Three dimensional N-doped graphene-CNT

networks for supercapacitor. *Chem. Commun.* 49, 5016-5018 (2013).

11. Li, Z., Wu, D., Liang, Y., Fu, R. & Matyjaszewski, K. Synthesis of well-defined microporous carbons by molecular-scale templating with polyhedral oligomeric silsesquioxane moieties. *J. Am. Chem. Soc.* 136, 4805-4808 (2014).
12. Xu, F. et al. Facile synthesis of ultrahigh-surface-area hollow carbon nanospheres for enhanced adsorption and energy storage. *Nat. Commun.* 6 (2015).
13. Xie, K. et al. Carbon Nanocages as Supercapacitor Electrode Materials. *Adv. Mater.* 24, 347-352 (2012).
14. Jin, Z.-Y., Lu, A.-H., Xu, Y.-Y., Zhang, J.-T. & Li, W.-C. Ionic Liquid-Assisted Synthesis of Microporous Carbon Nanosheets for Use in High Rate and Long Cycle Life Supercapacitors. *Adv. Mater.* 26, 3700-3705 (2014).
15. Zheng, X., Luo, J., Lv, W., Wang, D.-W. & Yang, Q.-H. Two-Dimensional Porous Carbon: Synthesis and Ion-Transport Properties. *Adv. Mater.* 27, 5388-5395 (2015).
16. Zheng, X. et al. Oriented and Interlinked Porous Carbon Nanosheets with an Extraordinary Capacitive Performance. *Chem. Mater.* 26, 6896-6903 (2014).
17. Puthusseri, D., Aravindan, V., Madhavi, S. & Ogale, S. 3D micro-porous conducting carbon beehive by single step polymer carbonization for high performance supercapacitors: the magic of in situ porogen formation. *Energy Environ. Sci.* 7, 728-735 (2014).
18. Qie, L. et al. Synthesis of functionalized 3D hierarchical porous carbon for high-performance supercapacitors. *Energy Environ. Sci.* 6, 2497-2504 (2013).
19. Huang, C. H. et al. Three-dimensional hierarchically ordered porous carbons with partially graphitic nanostructures for electrochemical capacitive energy storage. *Chemsuschem* 5, 563-571 (2012).
20. Chen, L.-F. et al. Synthesis of Nitrogen-Doped Porous Carbon Nanofibers as an Efficient Electrode Material for Supercapacitors. *ACS Nano* 6, 7092-7102 (2012).

21. Zhao, L. et al. Nitrogen-Containing Hydrothermal Carbons with Superior Performance in Supercapacitors. *Adv. Mater.* 22, 5202-+ (2010).

22. Li, W. et al. A Self-Template Strategy for the Synthesis of Mesoporous Carbon Nanofibers as Advanced Supercapacitor Electrodes. *Advanced Energy Materials* 1, 382-386 (2011).

23. Chen, W., Zhang, H., Huang, Y. & Wang, W. A fish scale based hierarchical lamellar porous carbon material obtained using a natural template for high performance electrochemical capacitors. *J. Mater. Chem.* 20, 4773-4775 (2010).
